# Case Report: Patient-specific three-dimensional printing and computational fluid dynamics in the planning of transcatheter aortic valve replacement for quadricuspid aortic valve

**DOI:** 10.3389/fcvm.2025.1555718

**Published:** 2025-07-08

**Authors:** Yujing Zhang, Dongxu Cai, Wei Wang, Hanyan Du, Jiwei Gu, Xiaodong Li

**Affiliations:** ^1^ Department of Cardiovascular Surgery, General Hospital of Ningxia Medical University, Yinchuan, China; ^2^ Graduate School, Ningxia Medical University, Yinchuan, China

**Keywords:** transcatheter aortic valve replacement, quadricuspid aortic valve, aortic regurgitation, computational fluid dynamics, three-dimensional printing

## Abstract

**Background:**

Performing transcatheter aortic valve replacement (TAVR) in patients with high-risk quadricuspid aortic valve (QAV) may be feasible, but uncertainties remain regarding the development of a comprehensive procedural plan and predicting the outcomes.

**Case summary:**

We report a case of a 70-year-old patient with a high-risk (EuroSCORE II: 11.2%) QAV (type B) and severe aortic regurgitation (regurgitant jet area measuring 9.8 cm^2^). To avoid high-risk surgery, we selected a 29-mm J-Valve for the transapical TAVR without the occurrence of paravalvular leak based on a patient-specific 3D printed model. Computational fluid dynamics simulations were performed to evaluate the hemodynamic parameters pre- and post-TAVR and showed that the trans-aortic valve pressure gradient decreased from 4.7 mmHg to 3.5 mmHg, the peak trans-aortic velocity decreased from 1.02 m/s to 0.89 m/s, and the low wall shear stress area was increased from 18.92 cm^2^ to 19.15 cm^2^. These findings suggest the effectiveness of the TAVR procedure. Based on the simulation results, the procedure was successfully implemented, leading to an improvement in the patient's clinical status.

**Conclusion:**

Three-dimensional printing and computational fluid dynamics simulations may be valuable tools for planning, assessing procedural outcomes, and evaluating risks in TAVR procedures for patients with QAV.

## 1 Introduction

The quadricuspid aortic valve (QAV) is a rare congenital malformation of the aortic valve (AV), with an incidence of less than 0.05% (1, 2). As the condition progresses, QAV is frequently associated with increasingly severe aortic regurgitation (AR) and may also lead to dilation of the ascending aorta or the development of aortic stenosis (AS) (1). Limited research and case reports indicate that over half of patients with QAV have reached a stage necessitating medical intervention (3). However, due to the rarity of QAV, there are no established protocols or robust evidence to guide its treatment. Surgical replacement or repair is currently the most common approach for managing severe AS or AR associated with QAV (4). Nevertheless, there is a notable absence of comprehensive data on surgical outcomes and long-term clinical results, and these procedures may increase the risk of complications and mortality, particularly in high-risk patients (1–5).

In recent years, a growing body of evidence has established transcatheter aortic valve replacement (TAVR) as a revolutionary technique that has transformed the treatment of the full spectrum of AV diseases (6, 7). This includes demonstrating safety and feasibility in patients with bicuspid AV. However, relevant randomized controlled trials have excluded patients with QAV. Only a limited number of case series and report outcomes have demonstrated favorable therapeutic results in patients with QAV treated with TAVR (5, 8, 9).

Cardiovascular three-dimensional (3D) printing technology has emerged as an invaluable tool, offering precise pre-procedural guidance for transcatheter interventions in structural heart disease, particularly in assessing device feasibility for complex and rare cases (5, 10). Additionally, computational fluid dynamics (CFD) simulations provide an effective approach for studying the effects of TAVR and can be used to investigate the interactions between pathological patterns and the implanted device (11–13). In this report, we present a patient with a QAV combined with severe AR. To optimize the planning process and evaluate potential enhancements in procedural outcomes, we utilized patient-specific 3D-printed models and CFD simulations to support pre-procedure planning.

## 2 Case presentation

A 70-year-old male patient with a history of hypertension and smoking was admitted to a local hospital one month prior, presenting with intermittent palpitations, severe dyspnea, and chest pain. Despite receiving optimal pharmacotherapy for symptomatic improvement—including metoprolol (1 × 100 mg), furosemide (2 × 100 mg), digoxin (1 × 10 mg), dapagliflozin (1 × 10 mg) and spironolactone (1 × 10 mg), the patient continued to exhibit limited exercise capacity and symptoms consistent with heart failure, classified as New York Heart Association functional class IV. Upon referral to our center, the patient's vital signs were as follows: blood pressure of 117/71 mmHg, heart rate of 69 beats per minute, respiratory rate of 19 breaths per minute, and oxygen saturation of 89% on pulse oximetry. Laboratory testing revealed an N-terminal pro-B-type natriuretic peptide level of 1,013 pg/ml.

**Figure 1:**
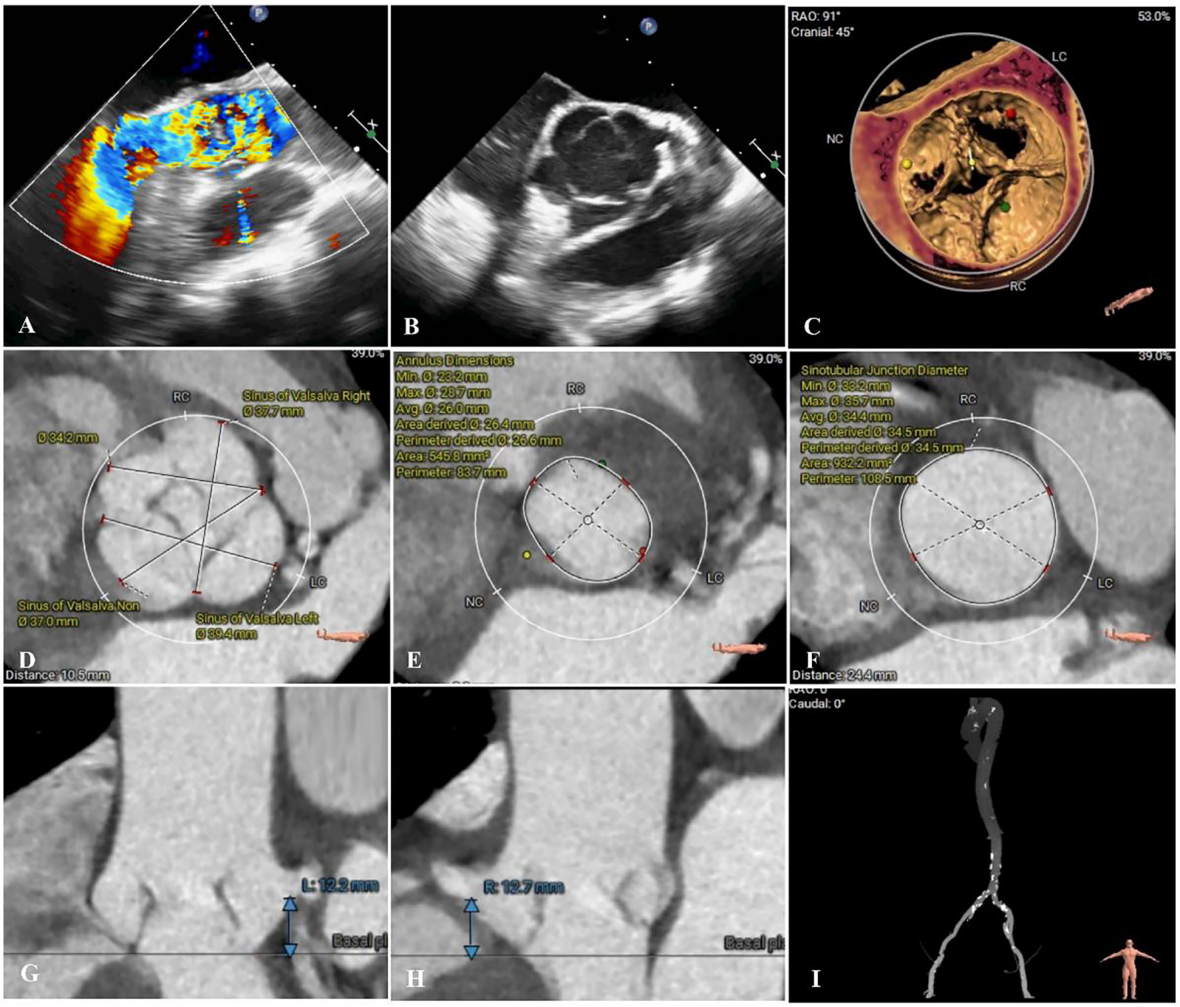
Preoperative imaging assessments. **(A,B)** Preoperative echocardiography revealed quadricuspid aortic valve and severe regurgitation (regurgitant jet area measuring 9.8 cm^2^). **(C–I)** Preoperative computed tomography angiography accessment. **(D)** Sinus of Valsalva diameters 37.0–39.4 mm; **(E)** Annulus diameter: 26.0 mm; **(F)** Sinotubular junction diameter 34.4 mm; **(G,H)** Left and right coronary ostia heights 12.2 and 12.7 mm.

Upon admission, vital signs were as follows: blood pressure of 118/63 mmHg, heart rate 78 b.p.m., respiratory rate 19 breaths per minute, and pulse oximetry 92% at ambient air. Transthoracic echocardiography revealed a congenital QAV with severe aortic regurgitation, mild aortic stenosis (regurgitant jet area measuring 9.8 cm^2^, flow velocity 1.86 m/s, trans-AV pressure 38 mmHg), and a left ventricular ejection fraction of 55% (Figures 1A, B). Computed tomography angiography (CTA) revealed the presence of a QAV (type B based on Hurwitz and Roberts classification). There was no significant valvular thickening or calcification observed. The mean diameter of the aortic annulus was measured at 26.6 mm, the ascending aortic diameter was 33.8 mm, and the sinotubular junction measured 34.4 mm. The mean left ventricular outflow tract diameter was 24.8 mm, while the left coronary artery measured only 12.2 mm, and the right coronary ostial height was 12.7 mm, which raises concerns about potential coronary artery occlusion (Figures 1C–I). Patients showed prohibit surgical risk (EuroSCORE II: 11.2%). After careful evaluation and in-depth discussion of all available treatment options and considering the patient's prohibitive surgical risk, the multidisciplinary cardiac team decided to perform TAVR. Informed consent was obtained from the patient for the procedure.

## 3 Patient-specific three-dimensional printing and computational fluid dynamics simulation

### 3.1 Patient-specific three-dimensional printing

**Figure 2:**
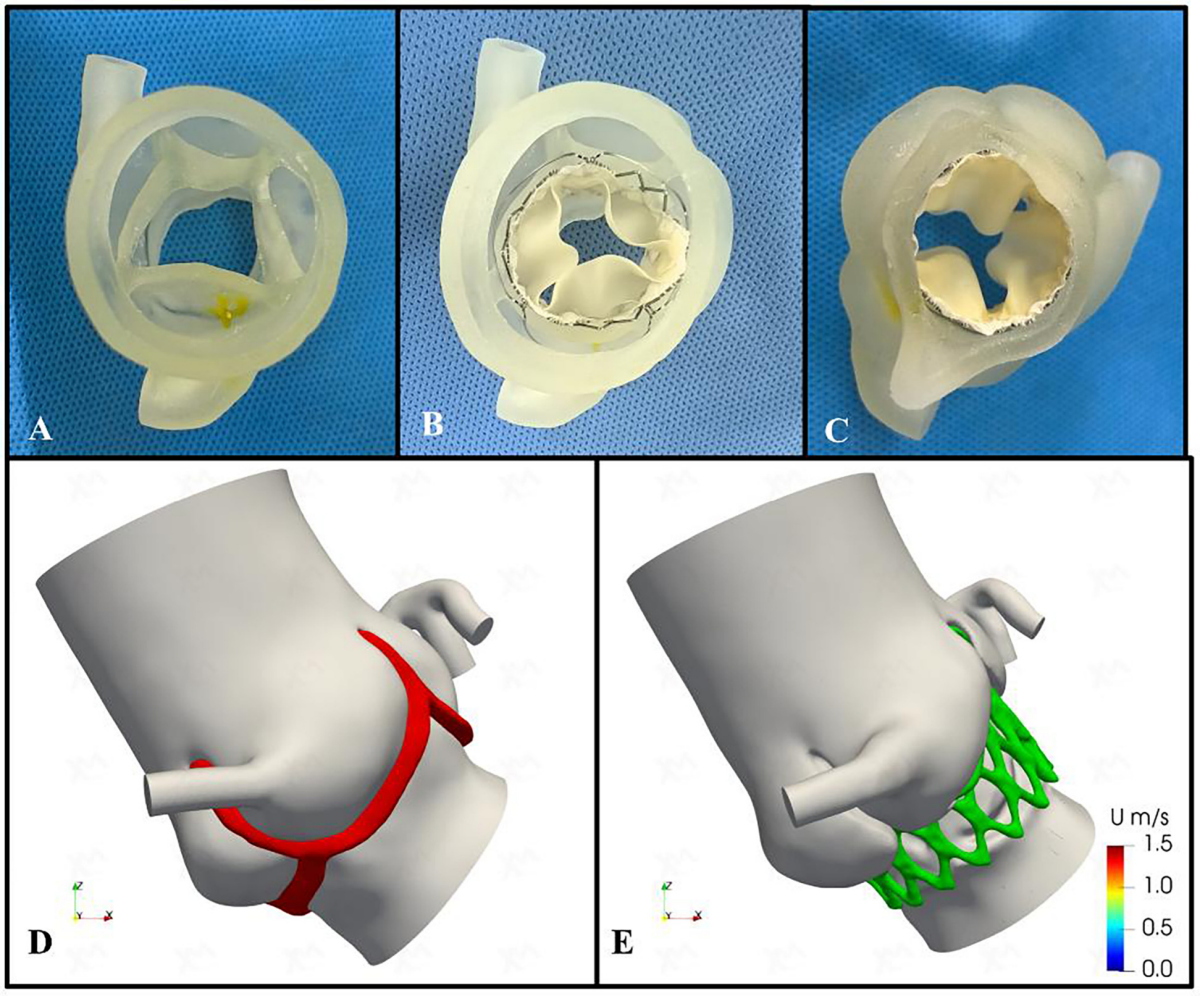
Preoperative patient-specific three-dimensional printing and computational fluid dynamics simulation. **(A)** Patient-specific three-dimensional printed aortic root model. **(B, C)** The preoperative simulation of implanting the 29-mm J-valve bioprosthesis valve. **(D,E)** Preoperative and postoperative computational fluid dynamics conceptual models. Red area indicates aortic valve; green area indicates bioprosthetic stent.

We conducted a comprehensive assessment of the aortic root structure using the patient's CTA data. The images were segmented using specialized software and exported in Standard Tessellation Language (STL) format. We subsequently post-processed the STL files using Materialise 3-Matic software (Leuven, Belgium) before importing the final STL files into the PolyJet 850 multi-material full-color 3D printer (Stratasys, Eden Prairie, MN, USA). Finally, we got a patient-specific aortic root 3D printed model (Figure 2A). During the bench-test simulation, we observed the anatomical structure of the 3D printed model, including the distribution of calcifications and the mobility of the valve leaflets. Considering the unfamiliarity with QAV and the uncertainty of circumferential measurements, we explored the implantation of different sizes and types of transcatheter heart valve (THV). However, due to the large size of the aortic annulus, there is an increased risk of paravalvular leakage (PVL) or even device displacement following implantation. Additionally, the procedure carries the potential risk of coronary artery obstruction.

Therefore, we assessed the feasibility of implanting a self-expanding THV, the J-Valve (Jiecheng Medical Technology, Suzhou, China), for treating pure AR and simulated the implantation process. The evaluation confirmed that the device's three positioning keys could be successfully implanted in the aortic sinus, securely clamping the three equal-sized leaflets while excluding the supernumerary leaflet. The three “U-shaped” locating keys were folded into three “long oval” shapes to facilitate accurate positioning. Additionally, the bioprosthetic valve could be fully deployed, achieving device stability without evidence of PVL (Figures 2B,C).

### 3.2 Computational fluid dynamics simulation

In addition, we utilized the processed STL files to generate high-quality meshes of the aortic root and ascending aorta. These were used to simulate the preoperative valve implantation process and to quantify the postoperative hemodynamic environment of the aortic root using finite element modeling and CFD methods (Figures 2D,E). The open-source CFD software OpenFOAM version 2212 (OpenCFD Ltd.) was utilized for the numerical simulations. The post-processing of the results was performed using Paraview (Kitware Co., Ltd.). The spatial discretization of the simulation domain was carried out using the embedded mesh generation tool snappyHexMesh in OpenFOAM. The maximum grid size was limited to 0.24 mm in order to accurately capture the morphological features and ensure grid independence of the solution (the voxel size is 0.340 mm × 0.340 mm × 0.750 mm). All the meshes are generated by the OpenFOAM embedded mesh tool snappyHexMesh and the major part of the mesh is composed by hexahedron. The objective of this CFD simulation was to assess the hemodynamic status during the cardiac systole and to evaluate the normality of blood ejection through the AV. To this end, the left ventricular outflow tract was used as the volumetric flow inlet, with a cardiac output of 4.8 L/min measured preoperatively via transthoracic echocardiography. The proximal ascending aorta and coronary arteries were defined as the pressure outlets (the systolic pressure is applied). For the numerical simulations, the aortic root complex and vessel walls were considered rigid, simplifying their movement during the cardiac cycle. Due to the lack of available data on the material properties of the AV and vessel structures, a fluid-structure interaction (FSI) is not applicable for this study. Therefore, a more feasible steady-state simulation was performed using the Semi-Implicit Method for Pressure-Linked Equations (SIMPLE) algorithm, which was based on the systolic geometry. To determine whether a laminar or turbulent flow model should be selected, we performed an approximate evaluation of the Reynolds number as follows:

In which *ρ*, u, d, and *μ* represent for blood density (1,060 kg/m^3^), velocity (1 m/s), characteristic diameter (2 cm, 0.02 m), and blood viscosity (3.8 × 10^−3^ Pa* s). The Reynolds-averaged Navier-Stokes equations (RANS) are selected for the mathematical calculation of turbulence flow at AV region. The time step is set as 5 × 10^−4^ s and the convergence criteria are set as 1 × 10^−3^ for the pressure and 1 × 10^−4^ for the velocity and other fluid dynamic parameters. The CFD simulation results can provide a comprehensive set of hemodynamic parameters within the region of interest. From these results, we extracted the blood flow velocity at the AV orifice and the systolic transaortic AV pressure gradient to quantitatively assess the changes in local obstruction after the TAVR procedure. Another crucial aspect to consider is the risk of mural thrombus formation following the implantation of the stent prosthesis (14, 15). Previous studies have established a strong correlation between the occurrence of low wall shear stress (WSS) and the formation of local thrombosis. Moreover, changes in the WSS distribution, particularly the variation in low WSS regions, can be critical factors (16, 17). In this study, these WSS variations were identified and extracted during the post-processing phase for subsequent analysis and discussion.

**Figure 3:**
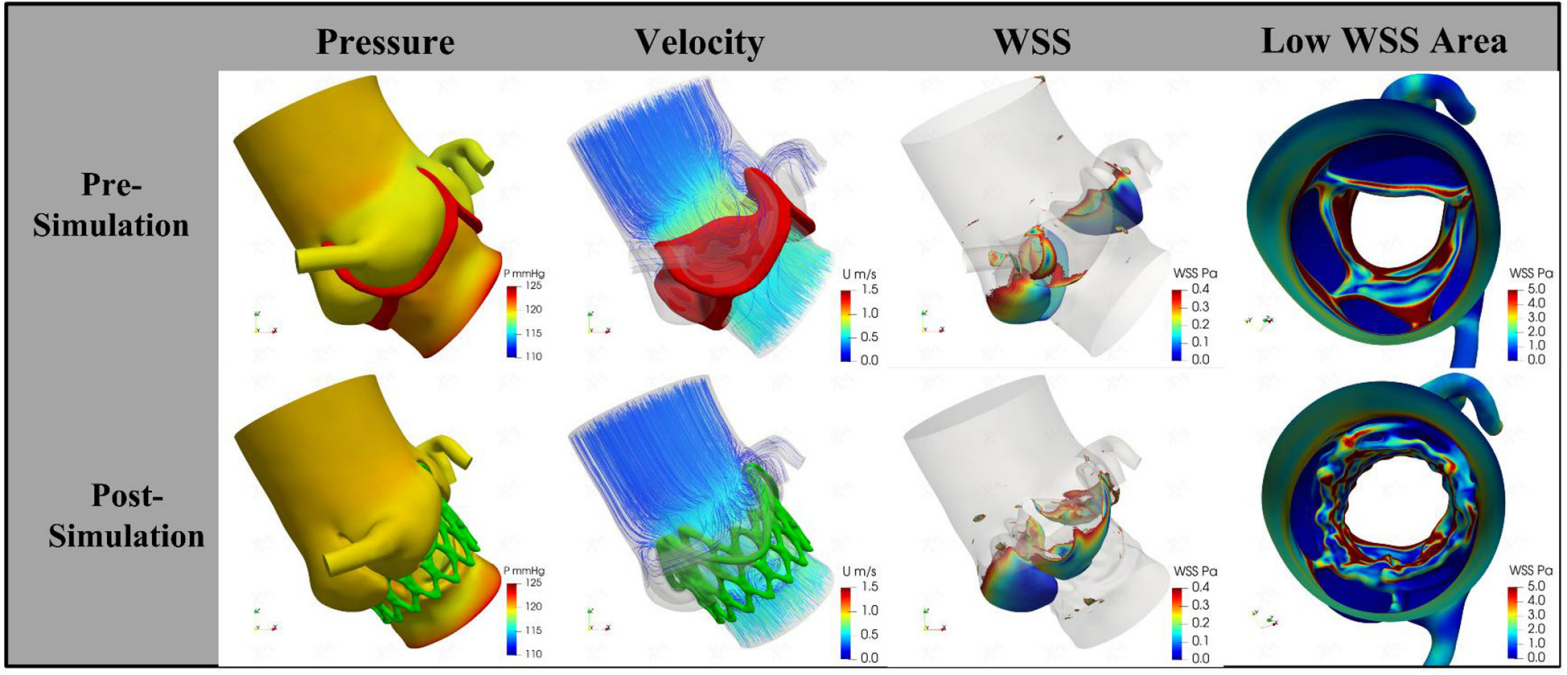
The change of pressure, velocity, and wall shear stress by procedural simulation. The trans-aortic valve pressure decreases from 4.7 mmHg to 3.5 mmHg; the velocity decrease from 1.02 m/s to 0.89 m/s; the low wall shear stress area increased from 18.92 cm^2^ to 19.15 cm^2^. WSS, wall shear stress.

Following the simulated implantation, the systolic hemodynamics of the aortic root were favorable, with local blood flow remaining smooth and without significant flow obstruction. The stent release did not result in substantial obstruction of coronary blood flow. The maximum blood flow velocity in the simulated area decreased from 1.02 m/s to 0.89 m/s, while the pressure dropped from 4.7 mmHg to 3.5 mmHg. The low wall shear stress area increased slightly from 18.92 cm^2^ to 19.15 cm^2^ (Figure 3). Based on these simulation findings, we decided to proceed with the implantation of a 29 mm J-Valve system via the transapical approach.

## 4 Transcatheter aortic valve replacement procedure

**Figure 4:**
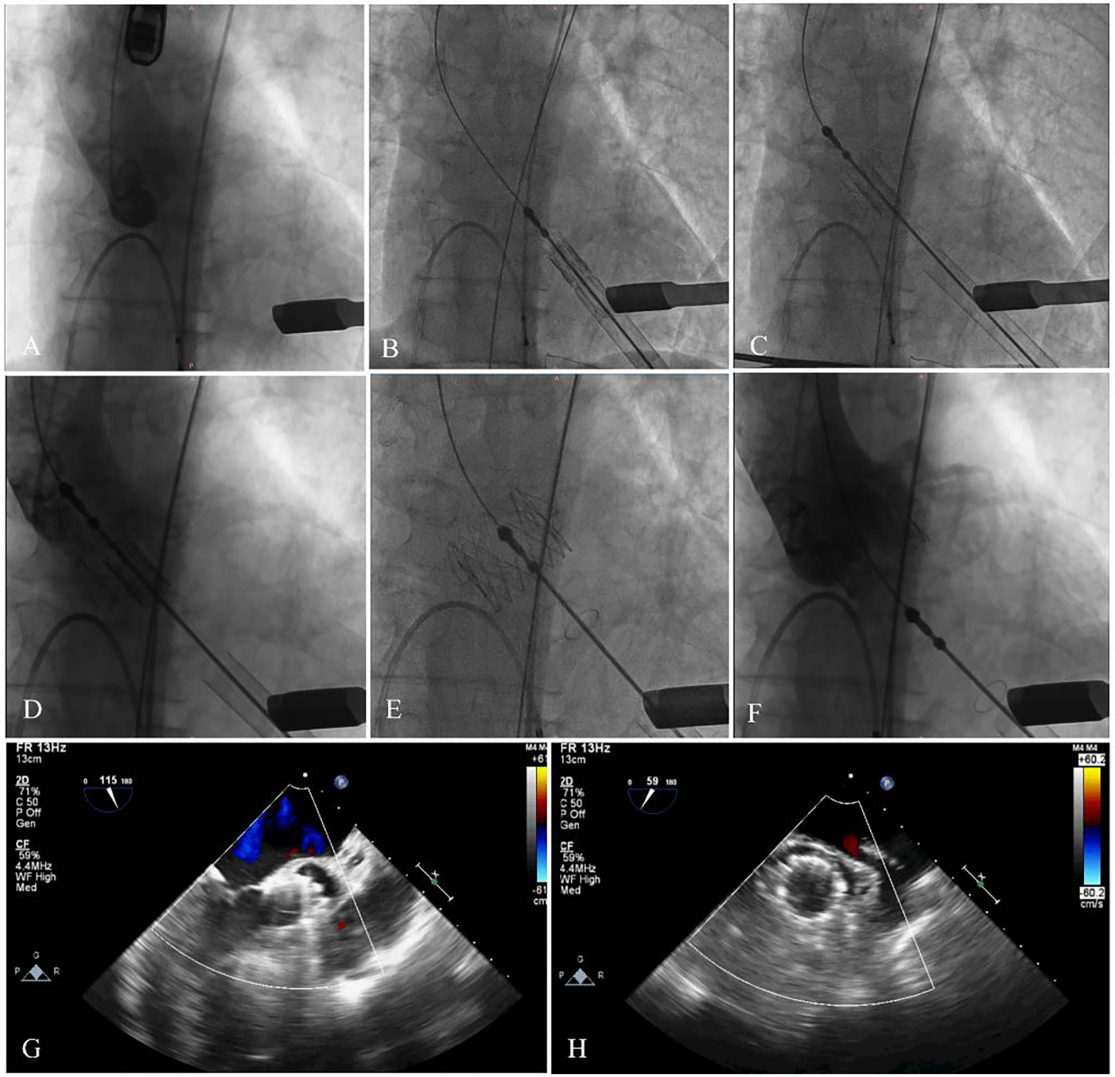
The main steps of the procedure. **(A)** Fluoroscopy showed the position of aortic root. **(B)** The delivery system was advanced via the transapical approach. **(C,D)** The delivery system was advanced. **(E,F)** The stent was fully released, and fluoroscopy revealed that the bioprosthesis was in a stable position and functioning well with no paravalvular leak and coronary artery occlusion. **(G,H)** Echocardiography showed no regurgitation, and excellent leaflet mobility.

The patient underwent TAVR under general anesthesia. A 6F pigtail catheter was introduced into the aortic sinus via the left femoral artery, and a temporary pacemaker was placed in the right ventricle through the left femoral vein. Valve pathology and apical positioning were evaluated using fluoroscopy and transesophageal echocardiography (TEE). The fluoroscopic angle was adjusted based on the preoperative CTA data to ensure that all three aortic sinuses were visualized simultaneously and aligned in the same plane. A small 4 cm incision was made along the fifth rib margin to expose the pericardium. An apical puncture was performed, and fluoroscopy confirmed the proper placement of a superstiff guidewire. Based on the preoperative assessment, a 29-mm J-Valve was selected, loaded into the delivery system, and advanced to the supra-annular position under fluoroscopic guidance. The locating keys were deployed into the aortic sinuses, and the lower end of the valve stent was aligned flush with the base of the three aortic sinuses. The valve was then released under rapid ventricular pacing at 140 beats per minute. Post-procedural TEE and fluoroscopy showed the successful elimination of regurgitation without PVL (flow velocity 0.76 m/s, trans-AV pressure 4 mmHg), preserved coronary artery patency, a stable valve stent position, and proper opening and closing of the valve leaflets. The apical purse-string sutures and intercostal incision were closed, and the patient was extubated immediately after the procedure (Figures 4A–F).

## 5 Follow-up

The patient was discharged from the hospital 7 days after the procedure. At the 1-month outpatient follow-up, the patient showed significant improvement, with improvement symptoms (New York Heart Association functional class II). Echocardiography showed a left ventricular ejection fraction of 61%, a mean pressure gradient of 2 mmHg, no PVL, and excellent leaflet mobility (Figures 4G,H).

## 6 Discussion

Nearly 50% of patients with QAV exhibit significant symptoms that necessitate aggressive intervention (1–4). Surgical replacement or repair has historically been regarded as the standard treatment for QAV associated with AS or AR (2–5). However, a significant proportion of elderly, multi-morbid, and frail patients are unable to tolerate the risks associated with conventional surgical intervention (4–8). In such cases, TAVR has emerged as a viable therapeutic option for symptomatic patients with AV disease, owing to its favorable safety and efficacy profile (6, 7). Despite the widespread adoption of TAVR in clinical practice, the feasibility of exploring available TAVR instrumentation for off-label applications is still being explored when faced with a larger number of patients presenting with anatomically diverse AV disease, including patients with QAV (1–9). The application of TAVR for the treatment of aortic valve disease in patients with QAV presents unique challenges regarding technical feasibility (5, 8–10). A thorough evaluation of the anatomical characteristics of the QAV is essential in determining the appropriateness of TAVR in these patients (5, 8–12). Because abnormal cusp distribution in QAV is associated with increased surgical complexity and a higher risk of perioperative complications, other coexisting structural abnormalities—such as intracardiac shunts, coronary artery anomalies, and the potential for PVL or valve displacement—should also be thoroughly assessed (5, 8–12).

Fukui et al. (18) reported their successful experience in performing TAVR for patients with QAV stenosis characterized by four unequal leaflets. They emphasized that interventional cardiologists should have a thorough understanding of the intricate anatomical relationships between the four cusps and the origins of any anomalous coronary arteries. This awareness is crucial to prevent improper transcatheter valve deployment and to mitigate the risk of coronary complications during TAVR in patients with QAV. In addition, unlike other types of AV, the unique anatomical and pathophysiological characteristics of QAV, coupled with limited surgical experience and other factors, present significant challenges (1–5). These issues require more in-depth research and clinical practice to effectively recognize and address them.

Currently, cardiovascular 3D printing offers unique advantages in guiding transcatheter techniques for treating structural heart disease (5, 10, 11). It enables the development of personalized surgical plans through iterative *in vitro* simulations and provides a visual assessment of potential complications arising from pathological structures (5, 10, 11). Oba et al. (10) performed TAVR for patients with QAV accompanied by severe AS. However, the risk of right coronary occlusion was assessed preoperatively by CTA, and simulation was performed using a patient-specific model of the aortic root. The simulation predicted no occlusion, and the intraoperative manipulation was consistent with the simulation results. Similarly, Liu et al. (5) obtained a 3D model of the aortic root to develop a TAVR plan and assess the risk based on the CTA data of five patients with QAV accompanied by severe AR. The TAVR procedure was performed according to the plan, and the results were consistent with the preoperative assessment.

In this case report, we present a patient who underwent TAVR for QAV accompanied by severe AR. A patient-specific 3D-printed model, based on cardiac CTA, was created to better understand the anatomy of the aortic root region. The model facilitated discussions on the type and size of the implanted THV and allowed for simulation of the surgical steps and potential complications. 3D printing enabled us to assess the compatibility of the device with the patient's specific anatomy. The simulations indicated that THVs with specific anchoring features were better suited to this unique structure. Additionally, the height of the coronary arteries on both sides was close to critical values, and the QAV exhibited longer leaflet heights and shallower cusp depths compared to a tricuspid AV. These anatomical factors increased the risk of coronary artery obstruction, even in the absence of other risk indicators. However, no coronary artery occlusion was observed after simulating the implantation of the selected device. Furthermore, no PVL was detected, and the risk of device displacement was found to be minimal.

In addition, the widespread use of advanced numerical computational programs and efficient pre- and post-processing equipment has facilitated the application of CFD in cardiovascular research (11–13). This has proven particularly valuable in analyzing the hemodynamic environment following device implantation and predicting surgical outcomes. Han et al. (11) demonstrated the role of using CFD simulation in predicting PVL in patients with QAV undergoing TAVR. They highlighted the importance of careful valve size selection and implantation depth to minimize the risk of PVL. Using CFD simulation, Dowling et al. (12) identified the likelihood of PVL after TAVR in patients with bicuspid and tricuspid AV. Based on these simulations, they were able to predict postoperative survival outcomes. Therefore, considering the interaction between the THV framework and the AV complex, this could significantly impact THV expansion, localization, and postoperative outcomes, particularly in patients with rare or complex aortic root lesions.

However, there are no definitive conclusions regarding the hemodynamic alterations following device implantation in patients with a QAV. Our combined use of CFD and imaging modalities enabled us to conduct patient-specific hemodynamic simulations, which helped us understand the mechanisms behind the abnormal blood flow profiles experienced by this patient postoperatively. The CFD simulation revealed that, in the postoperative period, both flow velocity and differential pressure in the aortic root were reduced. While the postoperative increase in low WSS area was relatively modest, this change suggests the need for long-term monitoring of local thrombosis formation, particularly in the context of stent implantation, which has been a key clinical concern following TAVR procedures (14–17). In contrast, the significant decrease in postoperative pressure and flow velocity demonstrated a substantial reduction in the risk of distal aortic vasodilation, providing a basis for the potential long-term survival benefits associated with TAVR. Additionally, the potential displacement of the implanted stent may alter the local hemodynamic conditions. Therefore, further studies are needed to clarify the relationship between the observed hemodynamic characteristics and the risk of adverse cardiovascular events. Consequently, we decided to implant a 29-mm J-Valve based on the simulation results. The postoperative TEE data provided hemodynamic measurements that validated the results of the CFD simulations. The patient demonstrated significant clinical condition improvement and was not observed to experience any adverse cardiovascular events.

## 7 Conclusions

This case demonstrates the feasibility of simulating the TAVR procedure in patients with a QAV using patient-specific 3D printing technology and CFD analysis. The simulation allowed us to evaluate the compatibility of the device with the patient's unique anatomy and provided a better foundation for surgical planning, as well as an assessment of potential outcomes and risks. This approach can help optimize TAVR in patients with complex aortic valve anatomy, such as QAV, and potentially improve procedural success and clinical outcomes.

## Data Availability

The original contributions presented in the study are included in the article/Supplementary Material, further inquiries can be directed to the corresponding author.
